# Intrawound low-dose vancomycin is superior to high-dose in controlling the risk of wound dehiscence in spine surgeries

**DOI:** 10.1097/MD.0000000000033369

**Published:** 2023-04-14

**Authors:** Ahmed M. Sonbol, Ayman M. Baabdullah, Mohamed Awad A. Mohamed, Farid N. Kassab

**Affiliations:** a Musculoskeletal Center of Excellence, International Medical Center, Jeddah, Saudi Arabia.

**Keywords:** culture-negative seroma, intrawound vancomycin, sterile seroma, surgical site infection, vancomycin powder, wound dehiscence

## Abstract

Wound complications in spine surgeries are common and serious. This study aimed to determine the risk of wound dehiscence with a low-dose of intrawound vancomycin compared to that with a high-dose and no-vancomycin and its effectiveness in the prevention of surgical site infection. Patients were categorized into 3 groups. The first group did not receive any intrawound vancomycin. In the second, patients received a high-dose of vancomycin (1 g). The third group included patients who received a low-dose of intrawound vancomycin (250 mg). Patient demographics, clinical data, and surgical data were also collected. Multivariate linear regression analysis was used to examine factors associated with dehiscence or infection. Of the 391 patients included in our study, 56 (14.3%) received a high-dose of intrawound vancomycin, 126 (32.2%) received a low-dose, and 209 (53.5%) did not receive any treatment. The overall incidence of wound dehiscence was 6.14% (24 out of 391 patients). Wound dehiscence was significantly higher (*P* = .039) in the high-dose vancomycin group than in the low-dose vancomycin group. The overall incidence of postoperative infection was 2.05% (8 patients) and no statistically significant differences were observed between the low-dose and high-dose vancomycin groups. Patients with higher body mass index were more likely to experience wound dehiscence and postoperative infection, irrespective of the dose of vancomycin used. The use of low-dose intrawound vancomycin (250 mg) resulted in less wound dehiscence compared with high-dose vancomycin. Further trials are required to evaluate the effectiveness of the low-dose in preventing postoperative infections.

## 1. Introduction

Wound infection is a common complication following posterior spine surgeries, with an incidence ranging from 0.7% to 11.9%.^[1–3]^ It is a serious complication and may require aggressive intervention with significant increase in cost.^[4–6]^ Certain wound infection prevention techniques are supported by high levels of evidence and have achieved broad acceptance (e.g., timely administration of antibiotics, maintenance of euglycemia). Other approaches, such as the use of intrawound antibiotics, are supported by less compelling data.^[7,8]^ Intrawound vancomycin powder has been used as prophylaxis against surgical site infections in spine surgery with promising results.^[9–11]^ Although it is a safe method systemically,^[12–14]^ wound dehiscence and formation of sterile seroma have been reported in the literature with this modality.^[15–17]^ Therefore, some reports have suggested to study the dose of local vancomycin powder administered in the wound.^[18–20]^ This study aimed to analyze the risk of wound dehiscence with low-dose (250 mg) intrawound vancomycin compared to that with a high-dose (1 gram) and its effectiveness in the prevention of surgical site infection.

## 2. Patients and methods

This was a retrospective chart review of all patients who underwent posterior thoracic, lumbar, or sacral spine surgeries at a single institution by a single specialized spine surgeon from December 2009 to September 2016. Cases of infection as an indication for surgery and cancer cases were excluded, and institutional review board approval was obtained for this study (Number: 2019-04-105). Data were collected from electronic medical records and included age, sex, body mass index (BMI), diabetes mellitus, renal impairment, procedure performed (decompression with or without fusion, scoliosis correction, fracture fixation), instrumentation, number of levels operated, postoperative systemic antibiotic prophylaxis (≤ 24 hours, > 24 hours, more than 1 agent), intraoperative dural tear, use of systemic steroids postoperatively, use of drain postoperatively, intrawound vancomycin dose (not used, high-dose, low-dose), postoperative infection status, and wound dehiscence.

Patients were considered to have wound dehiscence if the wound had not healed after 2 weeks as assessed during outpatient follow-up with no signs of infection, erythema, hotness or purulent discharge, and negative wound swab culture, if taken.^[21]^ Postoperative infection was considered in patients who had signs and symptoms of infection and positive wound culture after the surgery.

All elective spine cases were admitted on the same day or the day before the operation, whereas fracture cases were admitted from the emergency department. Intravenous cefazolin (2 g) was used in all cases as preoperative antibiotic prophylaxis 30 minutes before skin incision. Intravenous vancomycin (1 g) or clindamycin (600 mg) was administered in cases of allergies. The surgeries were performed by a fellowship-trained spine surgeon. At the end of surgery, either decompression or instrumentation, copious irrigation with saline was performed. Vancomycin powder, if used, was then spread over the dura, paraspinal muscles, bony posterior elements of the spine, instrumentation (if present), and subcutaneous tissue. Subsequently, no further irrigation was performed, and wound closure commenced. All wounds were covered with sterile and waterproof dressings.

The primary outcome was the difference in wound dehiscence or infection among patients who received a low-dose of intrawound vancomycin, patients who received a high-dose, and patients who did not receive any. The secondary outcome was the identification of predictors of wound dehiscence or infection after spine surgery in all groups.

Patients were divided into 3 groups. The first group consisted of those without the administration of intrawound vancomycin. The second group included those who received a high-dose of intrawound vancomycin (1 g). The third group included patients who received a low-dose (250 mg) of vancomycin into the surgical wound.

Descriptive and exploratory statistical analyses were performed using IBM SPSS Statistics for Windows (version 20). Fischer exact and Pearson chi-square tests were performed for the multivariate data sets. Differences were considered statistically significant at *P* < .05.

Logistic regression was applied to estimate the parameters of the binary dependent variables.

A regression coefficient of *R* < 0.05 was considered statistically significant. Descriptive analyses were expressed as means and standard deviations for continuous variables, whereas ordinal data were expressed as percentages.

## 3. Results

A total of 391 patients were included in our study, of which 56 (14.3%) received high-dose intrawound vancomycin, 126 (32.2%) received low-dose vancomycin, and 209 (53.5%) did not receive any. A descriptive analysis of each group is presented in Table 1. The overall incidence of wound dehiscence was 6.14% (24/391). The distribution of these patients among the 3 groups is described in Tables 2 and 3. Wound dehiscence was significantly higher (*P* = .039) in the high-dose vancomycin group than in the low-dose vancomycin group. The low-dose vancomycin group had a lower percentage of wound dehiscence than the group that did not receive any vancomycin; however, this difference was not significant. The overall incidence of postoperative infection was 2.05% (8 patients), as shown in Tables 2 and 3. There was no statistically significant difference in postoperative infection between the low- and high-dose groups. Furthermore, the infection rate was comparable between the low-dose group and the group that did not receive vancomycin. The causative organisms and onset of infection are listed in Table 4. Logistic regression was performed to ascertain the effects of age, sex, BMI, number of levels operated, type of procedure, presence of instrumentation, use of postoperative prophylactic antibiotics, use of drain postoperatively, intraoperative dural tear, diabetes mellitus, renal impairment, and use of systemic steroids postoperatively on the likelihood of the patients having wound dehiscence or postoperative infection. Of all variables assessed, only BMI was statistically significant (*P* < .05). The model explained 4.0% and 22.2% (Nagelkerke R2) of the variance in wound dehiscence and postoperative infection, respectively.

**Table 1:** Patient, clinical, and surgical characteristics.

	Total N* (%)	High-dose vancomycin N* (%)	Low-dose vancomycin N* (%)	Vancomycin not used N* (%)
Number of patients	391	56 (14.3)	126 (32.2)	209 (53.5)
Age (yr)				
Mean (SD†)	49.8 (17)	52 (16)	52 (16)	47 (17)
Body mass index (kg\m^2^)				
Mean (SD†)	30.2 (6)	31 (8)	30 (6)	30 (6)
Gender				
Male	212 (54.2)	29 (7.4)	65 (16.6)	118 (30.2)
Female	179 (45.8)	27 (6.9)	61 (15.6)	91 (23.3)
Procedure				
Decompression	361 (92.3)	56 (14.3)	113 (28.9)	192 (49.1)
Fracture fixation	12 (3.1)	0	6 (1.5)	6 (1.5)
Scoliosis correction	18 (4.6)	0	7 (1.8)	11 (2.8)
Number of levels				
1	247 (63.2)	33 (8.3)	78 (20.0)	136 (34.8)
2	83 (21.2)	15 (3.8)	23 (5.9)	44 (11.3)
3	30 (7.7)	7 (1.8)	14 (3.6)	10 (2.6)
4	10 (2.6)	1 (0.3)	3 (0.8)	6 (1.5)
5	1 (0.3)	0	1 (0.3)	0
6	3 (0.8)	0	1 (0.3)	2 (0.5)
7	3 (0.8)	0	1 (0.3)	2 (0.5)
8	7 (1.8)	0	2 (0.5)	5 (1.3)
9	3 (0.8)	0	1 (0.3)	2 (0.5)
10	1 (0.3)	0	0	1 (0.3)
11	3 (0.8)	0	2 (0.5)	1 (0.3)
Instrumentation				
Yes	188 (48.1)	28 (7.2)	53 (13.6)	107 (27.4)
No	203 (51.9)	28 (7.2)	73 (18.7)	102 (26.1)
Postoperative antibiotic prophylaxis				
24 h or less	203 (51.9)	43 (11.0)	50 (12.8)	103 (26.3)
More than 24 h	164 (41.9)	11 (2.8)	68 (17.4)	92 (23.5)
More than 1 agent	24 (6.1)	2 (0.5)	8 (2.05)	14 (3.5)
Intraoperative dural tear				
Yes	11 (2.8)	0	6 (1.5)	5 (1.3)
No	380 (97.2)	56 (14.3)	120 (30.7)	204 (52.2)
Renal function				
Normal	377 (96.4)	53 (13.6)	123 (31.5)	201 (51.4)
Impaired	14 (3.6)	3 (0.8)	3 (0.8)	8 (2.1)
Use of systemic steroids postoperatively				
Yes	54 (13.8)	13 (3.3)	17 (4.4)	24 (6.1)
No	337 (86.2)	43 (11.0)	109 (27.9)	185 (47.3)
Diabetes mellitus				
Yes	106 (27.1)	22 (5.6)	37 (9.5)	47 (12.0)
No	285 (72.9)	34 (8.7)	89 (22.8)	162 (41.4)
Use of drain postoperatively				
Yes	169 (43.2)	30 (7.7)	74 (18.9)	65 (16.6)
No	222 (56.8)	26 (6.7)	52 (13.3)	144 (36.8)

* N: number.

† SD: standard deviation.

**Table 2:** Wound dehiscence and infection in the low-dose group compared with those in the no-vancomycin group.

	Low-dose vancomycin n*= 126 (%)	Vancomycin not used n*= 209 (%)	*P* value	Odds ratio	95% CI
Wound dehiscence	4 (3.17)	13 (6.63)	.306	1.96	0.63–6.14
Wound infection	1 (0.79)	6 (2.87)	.262	3.62	0.43–30.39

CI = confidence interval.

* n: number.

**Table 3:** Wound dehiscence and infection in the high- vs low-dose groups.

	High-dose vancomycin n*= 56 (%)	Low-dose vancomycin n*= 126 (%)	*P* value	Odds ratio	95% CI
Wound dehiscence	7 (12.50)	4 (3.17)	.034	3.94	1.11–14.00
Wound infection	1 (1.79)	1 (0.79)	.57	2.25	0.14–36.62

CI = confidence interval.

* n: number.

**Table 4:** Types of organisms causing surgical site infection.

	Group	Onset of infection (postoperative d)	Causative microorganism
Patient 1	Low-dose vancomycin	21	*Proteus mirabilis Escherichia coli* (extended spectrum beta-lactamase)
Patient 2	Vancomycin not used	31	*Pseudomonas aeruginosa*
Patient 3	Vancomycin not used	15	Methicillin-resistant *Staphylococcus aureus*
Patient 4	Vancomycin not used	9	*Staphylococcus aureus*
Patient 5	Vancomycin not used	22	Methicillin-resistant *Staphylococcus aureus*
Patient 6	Vancomycin not used	32	Methicillin-resistant *Staphylococcus aureus*
Patient 7	Vancomycin not used	13	Methicillin-resistant *Staphylococcus aureus*
Patient 8	High-dose vancomycin	32	*Staphylococcus epidermidis* Enterococcus species

## 4. Discussion

In multiple recent studies, the use of intrawound vancomycin for spine surgeries has been found to be an effective method of decreasing the incidence of postoperative infection.^[22–27]^ In the present study, we found a lower infection rate with the use of high and low-doses of vancomycin; however, this was not significantly different. Although this method is safe for systemic antibiotic-related complications,^[28]^ delayed wound closure and culture-negative seroma were observed by multiple studies.^[27–29]^ Belykh et al^[16]^ reported an incidence of up to 10% of surgical site infection and delayed wound healing in the group of patients who received intrawound vancomycin. The formation of culture-negative seromas occurred in 15 patients in a retrospective case series by Ghobrial et al^[29]^ of 981 patients at a single institution who received an intrawound vancomycin dose of 1 to 6 g (mean 1.13 g). In vitro, Liu et al^[30]^ exposed osteoblasts, myoblasts, and fibroblasts to different concentrations of vancomycin ranging from 1 mg\cm^2^ to 12 mg\cm^2^ for 1 hour and 48 hours and compared them to a control group. They found that continuous vancomycin exposure and higher doses exerted cytotoxic effects on all these cells. Vancomycin concentrations as low as 1 mg\mL would decrease the viability of fibroblasts to 45% as per Herten et al^[31]^, who found a cytotoxic effect on endothelial cells, vascular smooth muscle cells, and fibroblasts at a concentration of 100 mg\mL. Goldschmidt et al^[32]^ harvested human dura and assessed the effects of different concentrations of vancomycin (40, 400, and 4000 μg/mL) on cell count and morphology. Compared with controls, vancomycin-exposed cells demonstrated more dural cell death, inhibited growth, histologically smaller cytoplasm, and decreased pseudopodia formation, resulting in the inhibition of normal intercellular spindle arrangement. Furthermore, intrawound application of a high concentration of vancomycin may reportedly interfere with osteoblast migration, proliferation, and differentiation and, therefore, increase the risk of nonunion or pseudoarthrosis after fusion surgeries.^[33–36]^ This is supported by our finding of a significantly lower incidence of sterile wound complications with the use of 250 mg vancomycin than with higher doses. In contrast, our results did not show an increased risk of wound infection with a low dose of intrawound vancomycin. The low-dose vancomycin group had a lower incidence of wound infection than the other groups, but this was insignificant. To our knowledge, no previous study has evaluated the effectiveness of intrawound vancomycin concentration of <500 mg in spine surgery.^[37,38]^ Based on this, we and other authors therefore encourage more clinical trials for lower doses of intrawound vancomycin.^[39,40]^ The use of intrawound vancomycin may increase the incidence of gram-negative and polymicrobial postoperative infections in our study; however, it is under debate in the literature.^[41]^ BMI showed an association in our study with wound infection and dehiscence, which is consistent with the findings in the literature of an increased incidence of wound complications with higher BMI.^[42–44]^

## 5. Limitations

The design of this study as a retrospective, single surgeon, and single center is the main limitation, and this would necessitate the need for more randomized multicenter studies with larger populations for further evaluation and considerations. Different doses and regimens can be evaluated as well as different types of antibiotics can be studied for the assessment of the risk of wound dehiscence.

## 6. Conclusion

The use of intrawound low-dose vancomycin (250 mg) resulted in less wound dehiscence compared with other higher standard doses. We encourage larger, randomized, prospective trials using low concentrations of intrawound vancomycin to study its effectiveness in the prevention of postoperative infection.

## Author contributions

**Conceptualization:** Ahmed M. Sonbol, Mohamed Awad A. Mohamed, Farid N. Kassab

**Data curation:** Ahmed M. Sonbol, Ayman M. Baabdullah, Mohamed Awad A. Mohamed, Farid N. Kassab.

**Formal analysis:** Ahmed M. Sonbol, Ayman M. Baabdullah, Mohamed Awad A. Mohamed, Farid N. Kassab.

**Methodology:** Ahmed M. Sonbol, Ayman M. Baabdullah, Mohamed Awad A. Mohamed, Farid N. Kassab.

**Project administration:** Ahmed M. Sonbol, Ayman M. Baabdullah, Mohamed Awad A. Mohamed.

**Resources:** Ahmed M. Sonbol, Mohamed Awad A. Mohamed, Farid N. Kassab

**Software:** Ahmed M. Sonbol.

**Validation:** Mohamed Awad A. Mohamed.

**Visualization:** Ahmed M. Sonbol.

**Writing – original draft:** Ahmed M. Sonbol, Ayman M. Baabdullah, Mohamed Awad A. Mohamed, Farid N. Kassab.

**Writing – review & editing:** Ahmed M. Sonbol, Ayman M. Baabdullah, Farid N. Kassab.
